# Simulation debriefing as part of simulation for clinical teaching and learning in nursing education: A scoping review

**DOI:** 10.1002/nop2.1426

**Published:** 2022-11-09

**Authors:** Liv Fegran, Wilma ten Ham‐Baloyi, Mariann Fossum, Olav Johannes Hovland, Joanne R. Naidoo, Dalena (R.M) van Rooyen, Ellen Sejersted, Nastasja Robstad

**Affiliations:** ^1^ University of Agder, Campus Kristiansand Kristiansand Norway; ^2^ Nelson Mandela University, Ocean Sciences Campus Port Elizabeth South Africa; ^3^ University of Agder, Campus Grimstad Grimstad Norway; ^4^ Nelson Mandela University, North Campus Port Elizabeth South Africa

**Keywords:** clinical teaching and learning, nursing, nursing education, scoping review, simulation debriefing, theoretical framework

## Abstract

**Aim:**

To map the evidence of the simulation debriefing phase in simulation activities of nursing education, to address and inform clinical teaching and learning in nursing.

**Design:**

A scoping review.

**Methods:**

A systematic review of literature published between 2008–2021 was conducted using CINAHL & ERIC, MEDLINE, EMBASE, APA PsycInfo, the Cochrane Library and JBI Evidence synthesis. Inclusion criteria were primary studies published in English on simulation debriefing at all levels in nursing education.

**Results:**

Of 140 included references, only 80% (*N* = 112) framed simulation debriefing theoretically either by specific theories/models or as a literature review of the topic. A variety of simulation debriefing methods were identified; however, debriefing methods were only described in 79% (*N* = 110) of the references. There appears to be a gap in consensus concerning the theoretical or methodological frameworks characterizing simulation debriefing in nursing education. The majority of studies (86%) were conducted at a bachelor's degree level (*N* = 121).

## 1 INTRODUCTION

Health professions education, and nursing education in particular, requires educators to adapt teaching and learning methods to produce skilled and competent health professionals equipped to ensure patient safety and improve patient outcomes (Kim & Yoo, 2020). Simulation as a teaching strategy could assist in producing skilled and competent health and nursing professionals because simulation allows for recurrent practice of technical and non‐technical skills until nursing students gain confidence (Kim & Kim, 2017). Further, simulation is an approach that can provide a realistic, safe environment while offering a solution to the intense competition for quality clinical placements for students (Eyikara & Baykara, 2017).

Simulation‐based education encompasses three phases: prebriefing, exposure to the simulation experience and debriefing (Chamberlain, 2015), the focus in this review is on the latter. Debriefing is an essential part of simulation‐based education and can be defined as a structured and guided process between people, after a simulated training session (Al Sabei and Lasater, 2016).

The focus of simulation debriefing (SD) is to provide feedback, analyse actions and encourage reflections to improve future performance. During SD, students are guided to reflect on the simulation experience under the supervision of the educator, who acts as the facilitator (Kim & Kim, 2017). For example, feedback about students' performance, errors and meta‐cognitive development is reflected upon (Kim & Kim, 2017). The debriefing phase is thus a critical activity for deep learning to take place through reflection and feedback, often regarded as a conversational period after the simulation experience. This phase is aimed at contributing to immediate change and improving future performance (Mulli et al., 2021). SD has shown to increase learners' knowledge, acquisition of skills, satisfaction in terms of valuing the nursing role, teamwork, communication experiences, self‐confidence, self‐reflection and enhancing student centeredness (Schober et al., 2019). To conduct high‐quality SD, the International Nursing Association for Clinical Simulation and Learning (INACSL^SM^) recommends that educators comply with five criteria. These criteria are as follows: simulation facilitators are individuals who (1) are confident in debriefing, (2) create an appropriate environment for debriefing that ensures confidentiality and use open communication, (3) are fully engaged in simulation, (4) align the content of simulation with simulation experiences and (5) use a theoretical framework to structure debriefing (INACSL Standards Committee, 2016).

A systematic review which aimed to identify SD frameworks and measures used to assess debriefing quality found that selection and training of simulation facilitators, together with using a debrief model and a debrief assessment, enhanced the quality of SD (Endacott et al., 2019). Further, the same review found a range of frameworks, strategies and models that could be used to underpin SD. For example, the Promoting Excellence and Reflective Learning in Simulation (PEARLS) framework is integrating three mutual educational strategies used during debriefing: (1) learner self‐assessment, (2) facilitating intensive discussion and (3) providing information in the form of directive feedback and/or teaching (Eppich and Cheng, 2015). The Debriefing for Meaningful Learning (DML) model emphasizes reflective thinking and the development of clinical reasoning in nursing students, focusing on six elements that facilitate distinct, yet integrated, thinking processes, including engage, explore, explain, elaborate, evaluate and extend, which are assessed through guided reflection and Socratic questioning (Dreifuerst, 2015). The Outcome Present State‐Test model is a synchronized information‐processing model of clinical reasoning that can be used as a teaching strategy by facilitators (Kuiper et al., 2008). The 3D model of debriefing includes three separate fragments: Defusing, Discovering and Deepening, which are performed after prebriefing or introduction (Zigmont et al., 2011). The Plus/Delta model of debriefing (Jeffries, 2010) emphasizes three main questions: *What went well? What would you like to change?* and *How to change?* Similarly, a variety of types or methods of SD exist: (1) facilitator‐guided post‐event debriefing, (2) self‐guided post‐event debriefing, (3) group discussions with or without videotaping, (4) computerized feedback debriefing, (5) individual versus group debriefing and (6) timing of debriefing either within the event or post‐event (Dufrene & Young, 2014; Sawyer et al., 2016).

In order for educators to effectively debrief, they should have the knowledge of basic debriefing concepts and educational strategies and the ability to facilitate complex debriefing interactions (Cheng et al., 2020), tailoring the timing of each debriefing (Schober et al., 2019) and leading the conversation, using questions and silence to facilitate learning (Macdiarmid et al., 2020). A structured debriefing guide, instrument or reflection tool can help educators and students in their learning environment and increase the students' clinical judgment, allowing for meaningful learning to take place (Al Sabei & Lasater, 2016; Reed, 2020). A recent integrative review identified the following debriefing methods and techniques used in nursing simulation (Nascimento et al., 2020): meaningful learning debriefing, debriefing based on principles of transfer of learning, the debriefing model of clinical reasoning and interprofessional debriefing, debriefing with good judgment and structured debriefing. Despite the comprehensive body of research exploring debriefing practices and methods, evidence‐based guidance for SD appears to be inconsistent and further exploration is required (Kim & Yoo, 2020; Lee et al., 2020).

Simulation debriefing is often used in the bachelor's or undergraduate levels of nursing education to develop deep learning through enhancing critical thinking skills and self‐efficacy among nursing students (Hall & Tori, 2017; Fey, 2014; Levett‐Jones & Lapkin, 2014). Although less frequent, there is evidence that SD can also be used in postgraduate or advanced levels of nursing education where it can be used to develop learner independence and adult learning, including a self‐directed, problem‐centred approach to learning and intrinsic motivation for learning (Spies & Botma, 2020).

As outlined above, SD is underpinned by a variety of theoretical frameworks, encompasses various methods and can be conducted at multiple levels of nursing education. Several authors have conducted reviews related to debriefing in simulation, but these have been limited to include randomized controlled trials only or solitarily focused on bachelor's degree nursing students (Dufrene and Young, 2014; Hall & Tori, 2017; Levett‐Jones & Lapkin, 2014; Sawyer et al., 2016). Mapping out all evidence about SD could provide readers with a more comprehensive overview and in‐dept understanding of the concept of SD, including its various theoretical underpinnings, methods used and its conduction at the various levels of nursing education. However, to date, no scoping review has been conducted in this regard.

## 2 DESIGN

This scoping review was conducted according to the Preferred Reporting Items for Systematic reviews and Meta‐Analyses extension for Scoping Reviews (PRISMA ScR) checklist (Appendix A) (Tricco et al., 2018).

## 3 ETHICS

Research Ethics Committee approval was not required for this scoping review.

## 4 METHODS

### 4.1 Aim

The aim of this scoping review was to map the evidence of the simulation debriefing phase in simulation activities at all levels of nursing education, to address and inform clinical teaching and learning in nursing.

### 4.2 Search methodology

The review was based on the methodology and guidance by Peters et al. (2015). The following steps guided the review: We (1) formulated the aim and review questions of the study; (2) searched for, retrieved, and selected the relevant research reports; (3) classified and synthesized the findings; and (4) presented the findings in tabular and narrative forms.

To develop the aim and research questions, the Population Concept Context (PCC) framework was used as follows in the protocol: Population: nursing educators or nursing students; Concept: SD; and Context: nursing education (Peters et al., 2020). The focused review questions were as follows:

1. What theoretical frameworks are used to describe and underpin the concept of SD?
2. What methods of SD are practised in nursing education?
3. What levels of nursing education are described in studies on SD?

### 4.3 Inclusion and exclusion criteria

To fulfil the aim of this review, primary studies published in English on SD at all levels of nursing education (i.e., undergraduate and postgraduate) were included.

Studies where nursing students participated in SD to improve clinical practice, and not to engage in scholarly activities related to clinical teaching and learning, were excluded. Studies including professions other than nursing (e.g., residents or other health professional students), non‐primary studies such as editorials, discussion papers, reviews, or guidelines, were also excluded. Studies in languages other than English were also excluded. Finally, references that were unable to be obtained in full text without payment were also excluded. The searches for studies were conducted between January 1 in 2008 and April 26 in 2021 in order to obtain the most recent evidence on a well‐studied topic.

### 4.4 Searching and retrieving research reports

A three‐step search strategy was conducted, inspired by the Joanna Briggs Institute (JBI) reviewer manual (Peters et al., 2020). The first initial broad search on words expressing simulation and debriefing was performed through CINAHL and MEDLINE (both through the EBSCOhost vendor), followed by an analysis of the text words contained in the title and abstract, and of the index terms used to describe the retrieved papers. The final search using the identified words for the concept “simulation debriefing” was undertaken in April 2021 across all the included databases – CINAHL & ERIC (EBSCOhost), MEDLINE, EMBASE and APA PsycINFO (Ovid), and the Cochrane Library and JBI Evidence synthesis – covering health and nursing educational literature. Because none of the included databases had an index term that matched the concept of SD, the search was performed on the words nearby, no more than 10 words between simulation and debriefing, in any field of the item in the database. The search strategy for MEDLINE (Ovid) simulat* adj11 debrief*.mp ^1^ was adapted to fit the other databases. The search was limited to publication year from 2008‐01‐01–2021‐04‐26 (Appendix B). After the search, the titles in the reference lists of identified studies were screened to obtain additional relevant studies. The studies from the reference lists were only considered for inclusion if they were published from 2008 and contained the word “debriefing” in the title. The retrieved items from the database search were uploaded to EndNote reference manager version 9.3 to deduplicate the retrieved reports, according to Bramer et al. (2016).

The unique references were then uploaded to Rayyan, a web‐based tool designed to help researchers with the screening and selection process (Ouzzani et al., 2016), for the initial screening of titles and abstracts. During the screening of titles and abstracts, the authors decided that if the word “debriefing” was mentioned in the title and/or abstract, the study should be included at this stage, and the full text should be read. The screening was performed by four of the authors paired in two teams of two, using a blinded process. After the screening, the blinding was turned off, and any disagreements between the two teams of authors were discussed in the research group until consensus for inclusion or exclusion was reached. The next step was to read the references in full text to assess whether the references fulfilled the study's objective and inclusion criteria, and all authors contributed by reading one‐eighth of the references. Any uncertainty about inclusion was discussed by the research team to obtain consensus on the inclusion or exclusion of references.

## 5 RESULTS

We retrieved 1961 unique references through the database search. After the initial screening of titles and abstracts, references were read in full text, and of these, 129 were included. Screening the reference lists of these included studies provided 11 more references to include, and in total 140 references composed the review.

**FIGURE 1:**
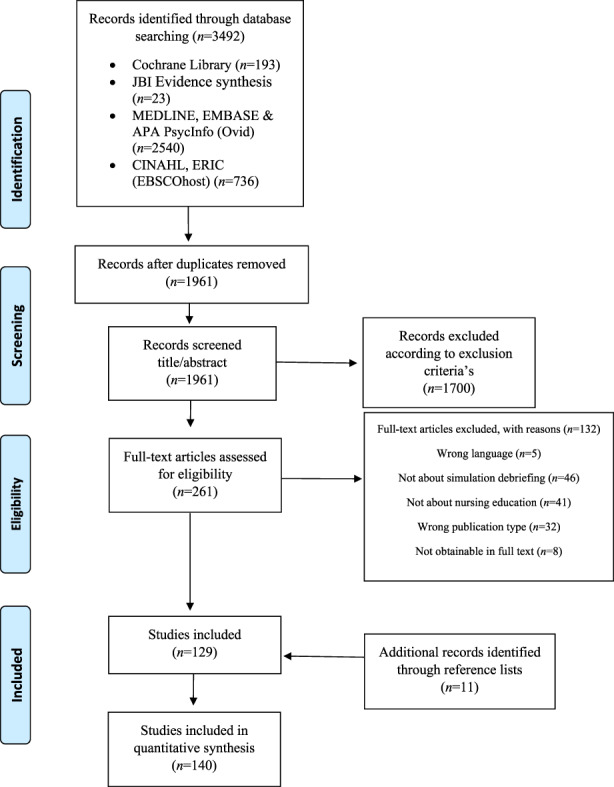
Prisma flow chart of literature search and selection process (Moher et al., 2009)

Characteristics of the included studies are presented in Table 1. The inclusion process is described in PRISMA (Figure 1) (Moher et al., 2009). The major reasons for exclusion were as follows: the studies were not about SD (*N* = 46), involved healthcare professions other than nursing (*N* = 41) or were not a primary study according to the introduction, methods, results, and discussion (IMRAD) structure (*N* = 32). Additional exclusion reasons were language other than English (*N* = 5) or references not obtainable free of charge (*N* = 8). Exclusion of references with reasons is presented in Appendix C.

**TABLE 1:** Characteristics of the included studies (=140)

Author	Year	Country	Theoretical framework for SD			Methods of SD					Level of education				
			Literature review	Not described	Described framework	Combination of methods	Facilitator/peer‐led	Group discussions	Structured	Not described	Associate degree	Bachelor's degree	Master's degree/	Combination of levels	Not described
Cantrell	2008	USA	x			x						x			
Kardong‐Edgren et al.	2008	USA		x						x		x			
Kuiper et al.	2008	USA			x				x			x			
Rush et al.	2008	Canada		x				x				x			
Clendinneng	2010	Canada	x							x			x		
Kaplan et al.	2010	USA		x				x				x			
Simones et al.	2010	USA		x				x					x		
Wotton et al.	2010	Australia	x				x					x			
Zulkosky	2010	USA			x						x				
Kaplan et al.	2011	USA		x						x		x			
Shinnick et al.	2011	USA	x									x			
Chronister et al.	2012	USA	x			x						x			
Dreifuerst	2012	USA			x	x						x			
Eikeland et al.	2012	Norway	x							x		x			
Hober	2012	USA	x							x		x			
Kaplan et al.	2012	USA	x							x		x			
Morse	2012	USA			x				x				x		
Reed	2012	USA	x			x						x			
Carvalho et al.	2013	Brazil			x					x		x			
Dufrene	2013	USA			x		x					x			
Husebo et al.	2013	Norway	x				x					x			
Mariani et al.	2013	USA	x						x			x			
Reed et al.	2013	USA			x	x						x			
Dusaj	2014	USA		x		x					x				
Fey	2014	USA	x						x			x			
Fey et al.	2014	USA			x					x		x			
Grant et al.	2014	USA	x			x						x			
Ha	2014	South Korea	x			x						x			
Kim et al.	2014	South Korea	x					x				x			
Mariani et al.	2014	USA	x						x		x	x			
Nevin et al.	2014	Ireland	x					x				x			
Roh	2014	South Korea	x				x					x			
Shortridge et al.	2014	UK	x			x						x			
Tosterud et al.	2014	Norway	x					x				x			
Weaver	2014	USA			x					x		x			
Bryant et al.	2015	USA	x					x				x			
Fey et al.	2015	USA			x					x		x			
Forneris et al.	2015	USA			x	x						x			
Gaylle	2015	USA	x			x						x			
Hayes et al.	2015	Australia		x						x		x			
Kanayama	2015	USA	x							x		x			
Morse	2015	USA			x				x				x		
Reed	2015	USA	x			x						x			
Ryoo et al.	2015	Korea	x						x			x			
Shea	2015	USA			x					x		x			
Tilton et al.	2015	USA	x				x					x			
Waznonis	2015	USA	x							x		x			
Barnard	2016	USA	x							x		x			
Bradley et al.	2016	USA			x				x			x			
Bussard	2016	USA			x			x				x			
Catanzaro	2016	USA			x			x				x			
Coutinho et al.	2016	Portugal			x				x			x			
Heise et al.	2016	USA		x						x		x			
Henry	2016	USA		x						x		x			
Kirkbakk‐Fjær et al.	2016	Norway	x							x		x			
Lestander et al.	2016	Finland	x						x			x			
Padden‐Denmead et al.	2016	USA	x						x			x			
Reed	2016	USA			x					x		x			
Roh et al.	2016	South Korea	x			x						x			
Seago	2016	USA	x							x	x				
Waznonis	2016	USA	x							x		x			
Abelsson et al.	2017	Sweden		x			x					x			
Bailey et al.	2017	USA		x			x					x			
Beroz	2017	USA		x						x		x			
Díaz‐Agea et al.	2017	Spain		x				x				x			
Howard	2017	USA		x				x				x			
Johnston et al.	2017	Australia	x			x						x			
Josephsen	2017	USA	x				x					x			
King et al.	2017	Canada		x						x		x			
Lavoie et al.	2017	Canada			x					x		x			
Luctkar‐Flude et al.	2017	Canada		x		x						x			
Reierson et al.	2017	Norway	x			x						x			
Roh et al.	2017	South Korea	x				x						x		
Rojas et al.	2017	USA			x				x						x
Rossignol	2017	USA	x			x						x			
Tutticci et al.	2017	Australia	x			x						x			
Bryant	2018	USA			x			x			x				
Gantt et al.	2018	USA			x	x						x			
Ha et al.	2018	South Korea		x		x						x			
Ha	2018	South Korea	x			x						x			
Haukedal et al.	2018	Norway			x		x					x			
Johnson et al.	2018	USA			x				x			x			
Kable et al.	2018	Australia	x						x			x			
Kang et al.	2018	South Korea	x			x						x			
Kim et al.	2018	South Korea		x		x						x			
Kirkbakk‐Fjær et al.	2018	Norway	x					x				x			
Loomis	2018	USA			x					x		x			
Nakayama et al.	2018	Japan		x					x			x			
Ostovar et al.	2018	Iran		x		x						x			
Tutticci et al.	2018	Australia		x		x						x			
Verkuyl et al.	2018	Canada			x	x						x			
Bae et al.	2019	South Korea			x					x		x			
Bortolato‐Major	2019	Brasil			x		x					x			
Colvin	2019	USA			x	x						x			
Coomes	2019	USA			x	x					x				
Gaylle	2019	USA		x		x						x			
Greco et al.	2019	USA		x					x			x			
Guimond et al.	2019	USA			x			x				x			
Johnston	2019	Australia	x			x						x			
Lavoie	2019	Canada			x	x								x	
MacLean et al.	2019	Australia			x			x					x		
Morley	2019	UK	x					x				x			
Nunes	2019				x		x					x			
Odreman et al.	2019	Canada			x	x						x			
Pinto	2019	USA			x		x				x				
Silva Vieira	2019	Brazil	x				x					x			
Verkuyl et al.	2019	Canada			x	x						x			
Verkuyl et al.	2019	Canada			x	x						x			
Zhang et al.	2019	Singapore	x			x						x			
Alhaj et al.	2020	USA			x	x								x	
Dudas	2020	USA			x	x						x			
Frandsen et al.	2020	Denmark			x	x						x			
Ha	2020	South Korea	x			x						x			
Jablonski	2020	USA			x	x						x			
Kennedy	2020	Qatar			x	x						x			
Kim	2020	Korea			x	x						x			
Kulju	2020	USA			x	x						x			
Lee	2020	South Korea			x	x						x			
Roca	2020	Spain			x		x					x			
Ross	2020	USA		x			x					x			
Secheresse	2020	France	x			x						x			
Sessions	2020	USA		x						x		x			
Verkuyl et al.	2020	Canada			x		x					x			
Verkuyl et al.	2020	Canada			x	x						x			
Verkuyl et al.	2020	Canada			x	x						x			
Weston	2020	USA			x					x		x			
Wilbanks	2020	USA		x		x							x		
Yeun	2020	Korea	x							x		x			
Zhang	2020	Singapore			x		x					x			
Zhang	2020	Singapore			x		x					x			
Atthill et al.	2021	Canada			x	x								x	
Campbell	2021	USA			x		x					x			
Escribano Sanchez	2021	Spain	x			x								x	
Ha	2021	South Korea	x			x						x			
Hu	2021	China			x		x					x			
Mac‐Kenna	2021	USA			x				x			x			
MacRae	2021	USA		x						x				x	
Oh	2021	South Korea			x			x				x			
Palominos	2021	Australia		x			x					x			
Rueda‐Medina	2021	Spain			x	x						x			

Characteristics of the included references (Table 1) revealed that most of the 140 included references were published in Northern America (*N* = 86). Several references were from Europe (*N* = 18), Asia (*N* = 22) and Oceania (*N* = 9), three studies were conducted in South America and two in the Middle East. Exploring the types of research designs used showed that most references (*N* = 79) used a quantitative design, 39 references used a qualitative design, and 22 references used a mixed methods research approach.

Simulation debriefing in nursing education has been well‐researched, as reflected by the number of publications since 2008. There was a peak in publications from 2014–2021 (*N* = 102), and as many as 19 of these were published since 2010. Simulation as a teaching strategy has been strongly developed in the last decade. As debriefing is considered a crucial part of simulation, this could explain the peak in publication numbers between 2014–2021 (Table 2).

**TABLE 2:**
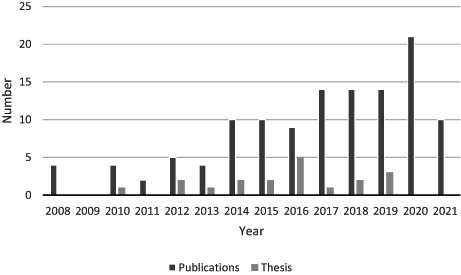
Number of publications (*N* = 140)

### 5.1 Theoretical frameworks describing and underpinning the concept of SD

The process of extracting data on how the concept of SD is described and underpinned in research related to nursing education appeared to be the most challenging step (Table 3). The research group had many discussions about whether the study was about debriefing or whether debriefing was a subordinate part of a study, for example, the study about how to increase self‐confidence by simulation and debriefing (Brown, 2008). More than half of the included references (*N* = 71) emphasized the concept of debriefing in the title, while others (*N* = 52) described the focus as simulation, or described both simulation and debriefing (16), and finally, one reference did not mention either simulation nor debriefing in the manuscript title. We approached concept clarification with a broader focus, describing how the authors had framed debriefing with theories, models and conceptual frameworks (Table 1). The research group pooled the findings of how the studies' presented their theoretical perspectives into three groups: (1) by debriefing theories, models and conceptual frameworks (*N* = *59*); (2) by a literature review of research on SD without choosing a specific theoretical framework for the study (*N* = 53). There was a gap in the studies' description of theoretical frameworks as 20 % (*N* = 28) lacked any description of a theoretical framework for SD.

**TABLE 3:** Theoretical framework for simulation debriefing (*N* = 140)

**Debriefing theories, models and conceptual frameworks**		**(*N* = 59)**
Debriefing for Meaningful Learning© DML (Dreifuerst)	14	
3D model of debriefing	6	
Experiential Learning Theory (Kolb)	9	
Reflective thinking exercise (Dewey, Schön)	5	
INASCL: Standards of Best; Practice℠: International Nursing Association for Clinical Simulation and Learning	3	
DASH©: Debriefing Assessment for Simulation in health care	2	
Debriefing with good judgment	2	
National League for Nursing/Jeffries simulation framework	2	
ResPoND: Reflective dEbriefing after a PatieNt Deterioration	2	
A three‐step approac	1	
Clinical Judgment Model and INASCL	1	
Gather–analyse–summarize	1	
INASCL and Promoting Excellence and Reflective Learning in Simulation (PEARLS)	1	
Mezirow's reflective thinking	1	
Multi‐moment debriefing model	1	
PEARLS and Experiential Learning Theory (Kolb)	1	
Plus delta	1	
Promoting excellence and reflective learning in simulation (PEARLS)	1	
Outcome present state‐test model	1	
RUST model of debriefing	1	
Steinwach's three stages	1	
The debriefing experience scale	1	
Transformative learning theory; defusing, discovery and deepening	1	
**Literature review of research on simulation debriefing**		**(*N* = 53*)***
**Not described a theoretical framework for simulation debriefing**		**(*N* = 28)**

### 5.2 SD methods

As depicted in Table 1, various SD methods were identified in the included references. A small number of the references used a form of structured SD. SD methods most frequently used were facilitator/peer‐led debriefing (*N* = 22), debriefing using group discussions/reflections (*N* = 18), followed by structured debriefing (*N* = 17). The term “structured debriefing” was not elaborated on in several of the included references, but was explained in some references (e.g., comprising reaction, reflection and summary) (Fey, 2014), or was part of a description, analysis or application (Ryoo and Ha, 2015). Almost one‐third of the references included a combination of SD methods (*N* = 53). Some *combined* two methods of debriefing, for example, individual written reflection and verbal group reflection (Lestander et al., 2016). Some studies *compared* different debriefing modes, for example, video‐assisted debriefing with verbal debriefing (Dusaj, 2014; Reed, 2012; Reierson et al., 2017), or peer‐led written debriefing versus instructor‐led oral debriefing (Ha & Lim, 2018). A total of 30 references did not document a specific SD method. However, some of these articles described a debriefing focus inappropriate for the aim of this study, for example, comparing various debriefing methods in different nursing education programs (Fey & Jenkins, 2015; Waznonis, 2016).

### 5.3 Level of education

One hundred and twenty‐one of the 140 references included participants from a bachelor's degree level of nursing education: 70 of these 121 studies described the year of the bachelor's degree (Table 1). The remainder of the references were conducted at an associate degree level (*N* = 6), master's degree, postgraduate or advanced level (*N* = 7) or a combination of levels of nursing education (*N* = 5). One reference did not describe the level of nursing education.

## 6 DISCUSSION

### 6.1 Theoretical frameworks used to describe and underpin the concept of SD

This scoping review provided a broad overview of the theoretical frameworks describing and underpinning SD, the SD methods and the education levels where SD in nursing education is used. SD theories, models and frameworks are important as they aid in the understanding on how debriefing is done (Cheng et al., 2020; Cheng et al., 2016). However, this review did not find consensus among included references on which debriefing theory, framework or model is most suitable for SD. Although the literature mentioned that the experiential learning philosophy underlies simulation education (Poore et al., 2014) this was not clearly reflected in our findings. Often, a theoretical framework describing and underpinning SD was lacking, or articles included literature reviews of research on simulation debriefing but did not mention a particular theoretical framework. Because debriefing is viewed as part of the process of simulation (Dreifuerst, 2009), it could therefore explain why theoretical frameworks on SD were not described as separate theories.

According to our review of the literature on SD in nursing education, few reviews summarized and tabulated SD theories, frameworks or models (Dufrene & Young, 2014). The Debriefing for Meaningful Learning model (Al Sabei & Lasater, 2016; Dreifuerst, 2012) was the most frequently used SD model in the references included in this review. This is unsurprising against the contextual application of the DML model, which was developed in North America, and most of the included studies for this review were from Northern America (*N* = 86). It seems, from the literature, that various approaches to SD can be used, including blended approaches (Eppich & Cheng, 2015), although these approaches may not always be underpinned by a SD theoretical framework. Further, according to Cheng et al. (2016), SD theoretical frameworks can have an impact on simulation‐based education.

### 6.2 Methods of SD practised in nursing education

Existing evidence suggests that structured SD methods are recommended to facilitate SD and should be performed at the end of the simulation session (INACSL Standards Committee, 2016; Hall & Tori, 2017; Lee et al., 2020). However, this review found various SD methods being used, including facilitator/peer‐led debriefing (which was most frequently used), structured debriefing, debriefing using guided group discussions/reflections and variations and comparisons of SD methods. Similar results were also found by another review (Cantrell et al., 2017) who indicated that debriefing can use an array of methods, conversational techniques and educational strategies to enhance the impact of SD. The variation in SD methods used may be of concern to promote best practices in SD. Some authors therefore promote the use of standards of best practice in simulation and SD, using standardized practices for SD, and the integration of systemic methods such as circular questions in SD, as such may mitigate simulation anxiety and provide structure for both students and the facilitator to optimize SD methods (Yockey & Henry, 2019).

### 6.3 Levels of nursing education described in studies on SD

With regard to educational levels, our review revealed that SD in nursing education is mainly done at a bachelor degree level. Simulation and SD in nursing education seems to be predominantly used in baccalaureate levels in order to build clinical skills and knowledge in a safe environment that is similar to that of a clinical placement (Lavoie et al., 2017). However, a national survey study about simulation debriefing practices in traditional bachelor degree nursing programs found issues related to the lack of evaluation of SD, poor student engagement, limited time and inadequate training among other things to pose challenges to SD in this context (Waznonis, 2015). These issues should be considered in order to optimize the use of SD at this level of nursing education. Further, simulation and SD of more complex situations at the postgraduate/advanced level could positively influence postgraduate students' knowledge acquisition, confidence and development of practical skills and enhancing teamwork and communication with other professions (Rød et al., 2021).

### 6.4 Implications for further research

In summary, although SD as part of simulation for clinical teaching and learning in nursing education has been well‐researched, the lack of consensus among the included articles on the theoretical descriptions and attributes underpinning SD and SD methods used, particularly at advanced or postgraduate levels of nursing education, is considered a gap to be explored for future research. A meta‐analysis could be conducted to understand how different theoretical frameworks or underpinnings influence proficiency in clinical learning among undergraduate students through SD. Furthermore, there is potential in investigating, for example through the use of randomized trials, which SD methods should be used best by educators when planning and conducting SD and the use of SD methods most suitable for different contexts and educational levels. Finally, further research through qualitative and quantitative studies should explore SD at advanced or postgraduate levels of nursing education as limited research at this level of education was found. Nurse educators and researchers may use the findings of this review to enhance SD practices and improve simulation experiences, and subsequently learning among nursing students.

### 6.5 Limitations and strengths

With the assistance of an experienced librarian, relevant databases were selected for a broad, comprehensive search on the topic. Using additional multidisciplinary databases such as Web of Science or Scopus, or hand search of relevant journals could have provided additional relevant studies on the topic. This limitation would also explain why eleven new studies were identified through the reference lists of the included references and not through searches in the databases. The included references comprised 19 theses and 121 journal articles. As many as eight theses were excluded because of full text required payment for access; thus, this exclusion criterion could be a limitation of the review. The timeframe for inclusion may have also limited our findings; however, only a small number of references is from before 2008. However, the large number of included references (*N* = 140) provided us with a broad picture of SD in nursing education between 2008–2021. As only references published in English were included, the five (*N* = 5) studies that were excluded during full‐text reading using Korean language could have provided data from one of the underrepresented continents. 28 of the references did not clearly describe the theoretical framework describing and underpinning SD, and 30 did not describe the SD methods used; thus, categorizing the findings was challenging. Majority (86%) of the included studies (*N* = 121) were conducted among undergraduate nursing programmes, where SD was especially vital as it was used to enhance the clinical knowledge and skills of students in a safe space before clinical placement.

By presenting the results in a table format, we have attempted to present the findings in a visual format to make the results clear and easy to follow. A strength of this review is that all the authors independently conducted the blinded screening to include the 140 references, using an available online web‐based application (Rayyan) and referencing tool (EndNote). The involvement of eight authors from Norway and South Africa could have restrained the process; however, this was rather a strength as this allowed us to develop our expertise on the topic and review methodology.

## 7 CONCLUSIONS

This scoping review has summarized data from 140 studies concerning SD in nursing education to determine the range of evidence on the topic. We found that simulation debriefing has been described in numerous studies as a crucial part of the simulation process used for clinical teaching and learning in nursing education and appears to be a highly valued method during nursing students' transition from scholarly activities to clinical practice. However, despite many publications from scientific journals and university repositories (Dissertations and Theses) in the last decade, there is a gap in consensus concerning the theoretical or methodological frameworks characterizing SD in nursing education, specifically at advanced or postgraduate levels, and further exploration is required.

## AUTHOR CONTRIBUTIONS

LF: Project administration. LF, WtHB, MF, OJH, JRN, DvR, ES, NR: Conceptualization. LF, WtHB, MF, OJH, JRN, DvR, NR: Methodology, data curation, review and editing. LF, WtHB, MF, OJH, NR: Writing. ES: Validation of literature search.

## FUNDING INFORMATION

This work was supported by the Network for Research on Professions (PRONETT) (grant number 68283/501620‐100) at the University of Agder, Norway.

## CONFLICT OF INTEREST

This article has not been published or presented elsewhere in part or in entirety and is not under consideration by another journal. We have read and understood your journal's policies, and we believe that neither the article nor the study violates any of these. There are no conflicts of interest to declare.

## Data Availability

Data will be available upon request from the corresponding author.

## Endnote

^1^
.mp (field code = multiple purpose). Adj11 – adjacency –allowing 10 words between the two words.

## APPENDIX A

### Preferred Reporting Items for Systematic reviews and Meta‐Analyses extension for Scoping Reviews (PRISMA‐ScR) Checklist

**Table:**

Section	Item	PRISMA‐ScR checklist item	Reported on page#
Title			
Title	1	Identify the report as a scoping review.	1
Abstract			
Structured summary	2	Provide a structured summary that includes (as applicable): background, objectives, eligibility criteria, sources of evidence, charting methods, results, and conclusions that relate to the review questions and objectives.	1
Introduction			
Rationale	3	Describe the rationale for the review in the context of what is already known. Explain why the review questions/objectives lend themselves to a scoping review approach.	4
Objectives	4	Provide an explicit statement of the questions and objectives being addressed with reference to their key elements (e.g., population or participants, concepts, and context) or other relevant key elements used to conceptualize the review questions and/or objectives.	5
Methods			
Protocol and registration	5	Indicate whether a review protocol exists; state if and where it can be accessed (e.g., a Web address); and if available, provide registration information, including the registration number.	No protocol
Eligibility criteria	6	Specify characteristics of the sources of evidence used as eligibility criteria (e.g., years considered, language, and publication status), and provide a rationale.	5
Information sources*	7	Describe all information sources in the search (e.g., databases with dates of coverage and contact with authors to identify additional sources), and the date the most recent search was executed.	6
Search	8	Present the full electronic search strategy for at least 1 database, including any limits used, such that it could be repeated.	6
Selection of sources of evidence†	9	State the process for selecting sources of evidence (i.e., screening and eligibility) included in the scoping review.	6
Data charting process‡	10	Describe the methods of charting data from the included sources of evidence (e.g., calibrated forms or forms that have been tested by the team before their use, and whether data charting was done independently or in duplicate) and any processes for obtaining and confirming data from investigators.	6
Data items	11	List and define all variables for which data were sought and any assumptions and simplifications made.	6
Critical appraisal of individual sources of evidence§	12	If done, provide a rationale for conducting a critical appraisal of included sources of evidence; describe the methods used and how this information was used in any data synthesis (if appropriate).	Not applicable
Synthesis of results	13	Describe the methods of handling and summarizing the data that were charted.	6
Results			
Selection of sources of evidence	14	Give numbers of sources of evidence screened, assessed for eligibility, and included in the review, with reasons for exclusions at each stage, ideally using a flow diagram.	6
Characteristics of sources of evidence	15	For each source of evidence, present characteristics for which data were charted and provide the citations.	7
Critical appraisal within sources of evidence	16	If done, present data on critical appraisal of included sources of evidence (see item 12).	Not applicable
Results of individual sources of evidence	17	For each included source of evidence, present the relevant data that were charted that relate to the review questions and objectives.	7
Synthesis of results	18	Summarize and/or present the charting results as they relate to the review questions and objectives.	7
Discussion			
Summary of evidence	19	Summarize the main results (including an overview of concepts, themes, and types of evidence available), link to the review questions and objectives, and consider the relevance to key groups.	9
Limitations	20	Discuss the limitations of the scoping review process.	10
Conclusions	21	Provide a general interpretation of the results with respect to the review questions and objectives, and potential implications and/or next steps.	11
Funding			
Funding	22	Describe sources of funding for the included sources of evidence, and sources of funding for the scoping review. Describe the role of the funders of the scoping review.	12

*Note*: From: Tricco AC, Lillie E, Zarin W, O'Brien KK, Colquhoun H, Levac D, et al. PRISMA Extension for Scoping Reviews (PRISMAScR): Checklist and Explanation. Ann Intern Med. 2018;169:467–473. doi: 10.7326/M18‐0850.

Abbreviations: JBI, Joanna Briggs Institute; PRISMA‐ScR, Preferred Reporting Items for Systematic reviews and Meta‐Analyses extension for Scoping Reviews.

## APPENDIX B

### Search strategi

Search strategy – 22‐26. April 2021

**Cochrane library**

Date: 22. April 2021

Search string:

(simulat* NEAR/10 debrief*):ti, ab, kw" with Publication Year from 2008–2021, in Trials

**Result: 193**

**CINAHL Plus with Full Text & ERIC (EBSCOhost).** (Link – Access restriction). Date: 26. April 2021

**Table:**

#	Query	Limiters/Expanders	Last Run Via	Results
S1	simulat* N10 debrief*	Limiters – Published Date: 20080101‐ Search modes – Boolean/Phrase	Interface – EBSCOhost Research Databases Search Screen – Advanced Search Database – CINAHL Plus with Full Text; ERIC	736 CINAHL: 676 ERIC: 60 deduplicated automatic exported to EndNote: 717 total exported to EndNote

**MEDLINE/EMBASE/APA PsycInfo (Ovid)**

Date: 22. April 2021

Database: Embase <1980 to 2021 Week 15>, Ovid MEDLINE(R) ALL <1946 to April 21, 2021>, APA PsycInfo <1806 to April Week 2 2021>. Search Strategy: Link to search

‐‐‐‐‐‐‐‐‐‐‐‐‐‐‐‐‐‐‐‐‐‐‐‐‐‐‐‐‐‐‐‐‐‐‐‐‐‐‐‐‐‐‐‐‐‐‐‐‐‐‐‐‐‐‐‐‐‐‐‐‐‐‐‐‐‐‐‐‐‐‐‐‐‐‐‐‐‐‐‐

1 (simulat* adj11 debrief*).mp. (2650)

2 limit 1 to year = “2008 ‐Current” (2540)

*3 remove duplicates from 2 (1683)*

Result total from the different databases before deduplication

**Table:**

limit 1 to year = "2008 ‐Current" Embase <1980 to 2021 Week 15> Ovid MEDLINE(R) ALL <1946 to April 21, 2021> APA PsycInfo <1806 to April Week 2 2021>	2540 1439 897 204

**Note**

adj# = adjacency/proximity:

- The ADJ operators finds two terms next to each other in the specified order.
- The ADJ1 operators finds two terms next to each other in any order.
- The ADJ2 operator finds terms in any order and with one word (or none) between them.
- The ADJ3 operator finds terms in any order with two words (or fewer) between them.
- The ADJ4 operator finds terms in any order and with three words (or fewer) between them, and so on

Search fields

- Search the .mp field, default field, that will include title, abstract, or any subject terms (index word)
- EMBASE. Default Fields for Unqualified Searches (MP): Searching for a term without specifying a field in Advanced search, or specifying .mp., defaults to the following ‘multi‐purpose’ (.mp.) fields for this database: ti, ab, hw, tn, ot, dm, mf, dv, kw, fx, dq.
- MEDLINE. Default Fields for Unqualified Searches (MP): Searching for a term without specifying a field in Advanced search, or specifying .mp., defaults to the following ‘multi‐purpose’ (.mp.) fields for this database: ti, ab, ot, nm, hw, kf, px, rx, ui, sy.
- PsycINFO. Default Fields for Unqualified Searches (MP): Searching for a term without specifying a field in Advanced search, or specifying .mp., defaults to the following ‘multi‐purpose’ (.mp.) fields for this database: ti, ab, hw, tc, id, ot, tm.

**JBI Evidence Synthesis**

**23 results** for: simulat* AND debrief* (all field) – limit: articles/all type

## APPENDIX C

### Exclusion with reasons

**Table:**

Reason for exclusion	*N* = 132	References
Not English language	5	Chae and Bang (2015), Jeong and Choi (2017), Ji and Kung (2013), Kim et al. (2009), Koh and Hur (2016)
Not about simulation debriefing	46	Adair et al. (2018), Al Camarero (2020), Aubin and King (2015), Barger (2019), Canclini et al. (2017), Craig et al. (2020), Anderson and Nelson (2015), Brown (2008), Burbach et al. (2016), Burbach et al. (2019), Call (2017), Chamberlain (2016), Coppens et al. (2018), Creed‐Hall (2017), Daley (2017), Da Silva (2019), Dumas et al. (2015), Fedko (2016), Foltz‐Ramos (2017), Fong (2013), Foronda et al. (2016), Gillan et al. (2013), Gillan et al. (2016), Gordon and Buckley (2009), Hallmark (2010), Hampson and Cantrell (2014), Holliday et al. (2020), Kada (2013), Knight et al. (2015), McClure et al. (2020), McKenna et al. (2011), Moura et al. (2020), Mulvogue et al. (2019), Nicholson (2012), Norwood (2008), Regan (2010), Reagan et al. (2019), Roy (2014), Rutledge et al. (2008), Shifrin et al. (2019), Widmar et al. (2019), Shuhaibar et al. (2010), Smiley (2019), Smith et al. (2013), Jeong et al. (2015), Yockey and Henry (2019)
Not about nursing education	41	Alanazi (2017), Ali (2015), Al‐Mehdi (2014), Alphonso et al. (2017), Altabbaa et al. (2019), Andersen et al. (2018), Ashmeade (2017), Attoe (2019), Aubin (2015), Bordelon et al. (2021), Brown (2018), Browning et al. (2016), Chamberland et al. (2018), Chris and Brett (2012), Colleen et al. (2021), Cooper et al. (2011), Cooper et al. (2012), Cripe (2020), Eggenberger et al. (2010), Evain et al. (2017), Fonseca et al. (2020), Fraser and McLaughlin (2019), Fraser et al. (2011), Gardner et al. (2017), Gibbs et al. (2015), Ginzburg et al. (2017), Goulding et al. (2020), Herlihy (2017), Hwang et al. (2018), Jaeger (2012), Johansson et al. (2017), Kim and Kim (2017), Kruger (2017), Leflore and Anderson (2009), Mak (2020), de Voest et al. (2019), Oikawa et al. (2016), Rudolph et al. (2014), Rueda‐Medina (2021), Tervajârvi et al. (2021), Timmis and Speirs (2015)
Wrong publication type (Not a primary study – IMRAD)	32	Beattie et al. (2010), Boyle (2015), Brohard and Moreland (2018), Brown (2011), Brown and Holt (2015), Brown and Watts (2014), cb56q19 (2019), Drost et al. (2019), Dreifuerst (2010), Fattouh et al. (2018), Fern (2014), Guinea et al. (2014), Harder et al. (2021), Harrison et al. (2019), Heise et al. (2018), Henneman and Cunningham (2005), Henneman et al. (2007), Hughey and Maaks (2019), Kelly et al. (2018), Kim et al. (2020), Lapum et al. (2019), Leavy et al. (2011), Mayville (2011), McClure and Gigliotti (2012), Nct (2020), Overstreet (2008), Reed (2013), Rose et al. (2020), Royle (2014), Turrise et al. (2019), Verkuyl et al. (2017), Waheed et al. (2018)
Not obtainable in full text:	8	Benhuri (2014), Cuerva et al. (2018), Harris et al. (2014), Brumfield and Leigh (2018), Martinez (2021), Olson (2013), Scudmore (2013), Willard (2014)
